# Simulation and Ergonomic Evaluation of Welders’ Standing Posture Using Jack Software

**DOI:** 10.3390/ijerph16224354

**Published:** 2019-11-07

**Authors:** Yongbao Zhang, Xiang Wu, Jingqi Gao, Jianwu Chen, Xun Xv

**Affiliations:** 1 School of Engineering and Technology, China University of Geosciences (Beijing), Beijing 100083, China; 2102180103@cugb.edu.cn (Y.Z.); gjq1118@cugb.edu.cn (J.G.); 2 China Academy of Safety Science and technology, Beijing 100083, China; cjw3000@126.com; 3 China Metallurgical Construction Research Institute (Shenzhen) Co., Ltd., Shenzhen 518040, China; 447183227@163.com

**Keywords:** welders’ standing posture, ergonomics, Jack software, adjustment strategy

## Abstract

Ergonomics research strives to make workers’ labor more efficient, safer, and more comfortable. Therefore, six digital humans and welding torch model were built and evaluated based on the Jack software in order to improve the ergonomics of welders’ standing postures. Three sets of standing welding actions were designed: walking, raising arm, and contracting arm. Through the Lower Back Analysis, Ovako Working Posture Analysis, Comfort Assessment, and Rapid Upper Limb Assessment, this paper evaluated the optimum range of the weight of the welding torch, the upper limb posture, and the neck posture of the welder. Firstly, the results show that Chinese welders should not use a welding torch with a weight of more than 6 kg when standing up. Secondly, for adult males in the 5th, 50th, 95th percentile of body size, the best operating distance is 321 mm, 371 mm, and 421 mm, respectively, and the best operating height is 1050 mm, 1100 mm, and 1150 mm, respectively; for females in the same percentiles, the optimal operating distance is 271 mm, 321 mm, and 371 mm, respectively, and the optimal operating height is 1000 mm, 1050 mm, and 1100 mm, respectively. Moreover, the horizontal and vertical rotation angle of the welder’s neck should not exceed 15° and 8.7°. The adjustment strategy not only has a positive effect on improving welders’ operational posture and preventing fatigue and injury to the welder, but it also develops research ideas for promoting safety from the perspective of ergonomics.

## 1. Introduction

Welders comprise a large occupational group that works long hours in forced postures [1]. Maintaining forced posture can cause early muscle fatigue [2], while it can lead to work-related musculoskeletal disorders (WMSDs) in the long-term or in severe cases [3]. Moreover, prolonged forced postures can lead to occupational injuries to workers [4], which will cause long-term physiological and psychological harm [5]. A survey of forest workers in New Zealand have found that physical fatigue might constitute a significant risk factor for accidents and injury [6]. Meanwhile, WMSDs are the most common occupational injury worldwide and the most common cause of long-term pain and disability in workers [5]. It is undeniable that full mechanization would be the best approach towards minimizing worker fatigue and injury, but, because of the high cost, ergonomic interventions for workers are still necessary and effective for smaller scale businesses [7]. Therefore, on the basis of the current situation regarding serious occupational hazards and safety, ergonomics research focused on welding posture in China can effectively protect people’s physical and mental health and prevent accidents.

From an ergonomic perspective, the evaluation of workers in different fields has already provided mature theories and a large number of research results. Some scholars have conducted a questionnaire survey on the health status of primary and secondary school teachers in Hong Kong, and found that, in addition to mental stress, work-related musculoskeletal disorders should also be valued [8]. On the basis of the Nordic Musculoskeletal Disorder Questionnaire, the results of ergonomics related to nurses have shown that poor working postures tend to cause WMSDs, and measures to prevent WMSDs in nurses have been proposed [9]. Other scholars have proposed starting from a training and management perspective to overcome or reduce the adverse effects of welding on the upper limbs by developing a training program for welders [1]. In addition, Francisco C and Edwin T analyzed the stress on the upper limbs during the work of the auto assembly welder. They proposed regularly adjusting the welding work site, which encourages the welder to frequently change posture and welding torch, for the purpose of reducing any occupational hazards [10], this program has had a positive effect on improving common occupational injuries. Similarly, the ergonomic study of office workers in the United States shows that, whether standing or sitting, for a long time, it will have an adverse effect on the lower back, and relaxation seems to be more effective in avoiding injury [11]. In 2017, Goncn et al. used computer software to conduct ergonomic studies on the working posture of wheeled mowers as well as evaluating the performance of the wheeled mowers [12]. Moreover, in terms of ergonomic visibility, Qiu Shiguang’s team evaluated the ergonomics of maintenance workers’ hand tool repair operations by writing a programming language to check whether there is any obstacle between the line of sight and the target part to determine whether it is visible or not [13]. Recently, Brazilian researchers conducted ergonomic evaluations of workers carrying two types of beer kegs and proposed ways for optimizing this [14]. It is not difficult to see that the study of ergonomics is roughly divided into two parts: the improvement of equipment and the improvement of working posture. Therefore, welding posture and welding torch performance in accordance with safety ergonomic requirements can increase welder productivity, reduce injuries and accidents, and enhance the economics of a business.

In the literature, most scholars use questionnaires or observations to explore and evaluate ergonomics. However, there are fewer studies that make accurate quantitative evaluations of human hazards. Some scholars have used simulation tools to quantitatively study a certain part of the human body, but this is not comprehensive. At present, there are few ergonomic studies on welders, and some of the models that were established in the related research have been oversimplified. Many research conclusions based on European body data do not reflect the true working state of Chinese welders. Therefore, further research is needed to break through the limitations of traditional research methods and validate and supplement existing results. Herein, the Jack digital human body simulation software is used to evaluate the posture of Chinese welders. In this study, a common standing welding posture was selected; this was done while using a safety ergonomic analysis method and using digital human body simulation technology to simulate the manual welding operation of welders. By studying the weight of the welding torch, the upper limb force of the welder, and the line of sight of the welder during the welding operation, an adjustment strategy related to the welding torch weight, the welder’s upper limb posture, and the welder’s neck posture are proposed. The results can improve the working conditions and working methods of Chinese welders and improve the health and safety conditions.

## 2. Research Methods

### 2.1. Digital Human Body Modeling

This study mainly used three-dimensional (3D) simulation technology to analyze the ergonomics of the standing welding operation while using a hand-held welding torch. The researchers selected the ergonomics analysis software Jack version 8.3 as a tool to model and import the digital human and welding torch, build the overall simulation scene of the welding operation, simulate the dynamic welding operation, and realize the operation and analysis of the action example. Four evaluation analysis modules for Lower Back Analysis (LBA), Ovako Working Posture Analysis (OWAS), Rapid Upper Limb Assessment (RULA), and Comfort Assessment (CA) in the Jack software were used to qualitatively and quantitatively analyze the weight of the welding torch and the welder’s posture during welding. The best welding torch weight and welding posture for maximum comfort and better working conditions were selected on the basis of the results of the evaluation.

### 2.2. Welding Environment Setting

**Figure 1:**
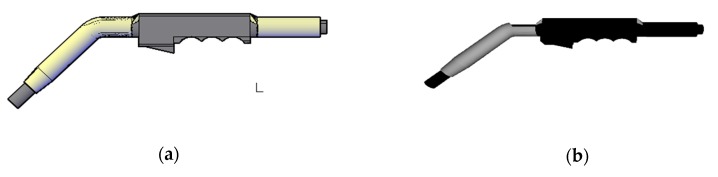
Three-dimensional (3D) model of the welding torch. (**a**) CAD welding torch model. (**b**) Jack torch model.

Three sets of standing welding action modules for walking, raising arm, and contracting arm were designed while using the Jack software to simulate the welding operation posture. The design process of the experimental simulation is as follows:

(1) the different percentiles of Chinese localized welder’s body size, as shown in Table 1 (the body size parameters are based on the Asian human body database in the Jack software, with reference to GB10000-88 Chinese Adult Body Size and GB/T13547-92 Workspace Human Body Size in anthropometric data), were used to create a Chinese localized welder body model;
(2) the welding torch model was introduced into the working environment, as shown in Figure 1. According to the welding torch positioning technology, the program written on the Jack Script secondary development platform was used to achieve the fit of the welding torch, the palm, and the solder joint;
(3) after the fit was completed, the human body control window was used to adjust the static posture of the person, including the hand, arm, shoulder posture, etc. The palm shafts of both hands add the weight and load of the welding torch to the hand of the model; and,
(4) the static posture was sequence-adjusted, spliced into dynamic behavior, a welding operation animation was created for the welding process, and the animation was classified into three motion modules. Data were collected for LBA, CA, OWAS, and RULA while completing the experimental animation.

### 2.3. Welding Torch Weight Setting

The welder’s process of holding a welding torch for welding operations is essentially that of a person lifting heavy objects. The Jack software can be used to evaluate the standing welding posture and analyze the body’s force through real-time observations when lifting the lower arm in the working environment. In this study, the lifting arm mainly evaluated the following dimensions:

- Lower back pressure
  The lower back pressure mainly indicates the force of the L5/L4 lumbar vertebrae [15]. In this study, the lower back pressure values were collected during the execution of the three welding action modules and compared with the database of The National Institute for Occupational Safety and Health (NIOSH) to determine whether each value was within the controllable range. According to the NIOSH database, a lower back disorder might occur when the lower back pressure exceeds 3400 N [16]. The higher the value, the greater the possibility and severity of lower back injury [17].
- Comfort value
  Comfort indicates the degree of hazard that is caused by a particular behavior and provides an optimized recommendation when the welder lifts the torch. The comfort assessment kit based on the Porter1998 database provides a corresponding human posture comfort rating. The relevant parameters of the virtual welding work were collected and converted into comfort values while using Formula (1). The best comfort value is 0, the comfort value is acceptable within 0–1, more than 1 needs to be improved, the higher the value, the lower the comfort [18].
  (1) $$CV=\left\{\begin{array}{c} \frac{\left|MD-OV\right|}{HV-MD},OV>MD\\ \frac{\left|MD-OV\right|}{MD-LV},OV<MD\\ 0,other \end{array}\right\}.$$
  *CV*: converted comfort value;
  *MD*: mode value;
  *OV*: original value;
  *HV*: highest value; and,
  *LV*: lowest value.
- Ovako Working Posture Analysis system
  The Ovako Working posture Analysis system can evaluate the ease of stretching of the back, and the upper and lower limbs, and can qualitatively and quantitatively analyze the practicality of the posture and the possibility of suffering from WMSDs [19]. It is possible to identify a posture that is harmful to the worker’s body and reduce the fatigue of the worker while using this analysis [20].

### 2.4. Upper Limb Posture Setting

During welding work, the welder might have to raise the arm, bend over, lean forward, etc., due to the difference in the position of the welding point and different weights of upper limb load, which might easily lead to discomfort and injury.

When evaluating the upper limb posture, in addition to the LBA, as mentioned earlier, RULA was also used. RULA assesses the risk of upper limb injury based on posture, muscle use, the weight of loads, and task duration and frequency. RULA gives a value that indicates the degree of intervention that is required to reduce the risk of an upper limb injury. Specifically, Level 1: Acceptable posture if not maintained or repeated for long periods (grand score 1–2); Level 2: Further investigation needed, may require changes (grand score 3–4); Level 3: Investigation, changes required soon (grand score 5–6); and, Level 4: Investigation, changes required immediately (grand score > 6) [7]. In short, the higher the RULA score, the stronger the discomfort of the upper limbs [21].

The arm (hand) function radius of rotation and the arm (hand) comfort zone height can describe and define the horizontal distance and vertical heights of the upper limb operations, respectively. The electric power industry standard that China issued in 1999: The ergonomic principles for the design of control centers, part 3, hand reach and zones of control (DLT 575.3-1999) stipulate the range of arm operation for sitting and standing positions. Arm (hand) function rotation radius, arm (hand) comfort operation area height are determined based on the standard of the male 5th percentile of body size. An additional evaluation of the arm (hand) function radius of rotation and the arm (hand) comfort zone height was required to measure and evaluate the corresponding stress in the lower back and upper limbs in order to study the comfortable operation space of the digital human with different percentiles and genders.

According to the DLT 575.3-1999, the 5th percentile of body size male arm (hand) has a minimum functional radius of rotation of 321 mm, the arm (hand) has a maximum functional radius of 610 mm, and the comfortable operating zone height ranges from 1050 mm to 1400 mm. Again, in males, the difference in arm length between the 5th, 50th, and 95th percentile is about 50mm; in the same percentile, the difference in arm length between different genders is approximately 50mm. Therefore, Table 2 and Table 3 show the calculation results of the arm (hand) function rotation radius range and the comfort operation area height range of the six different percentiles and different genders.

### 2.5. Neck Posture Setting

**Figure 2:**
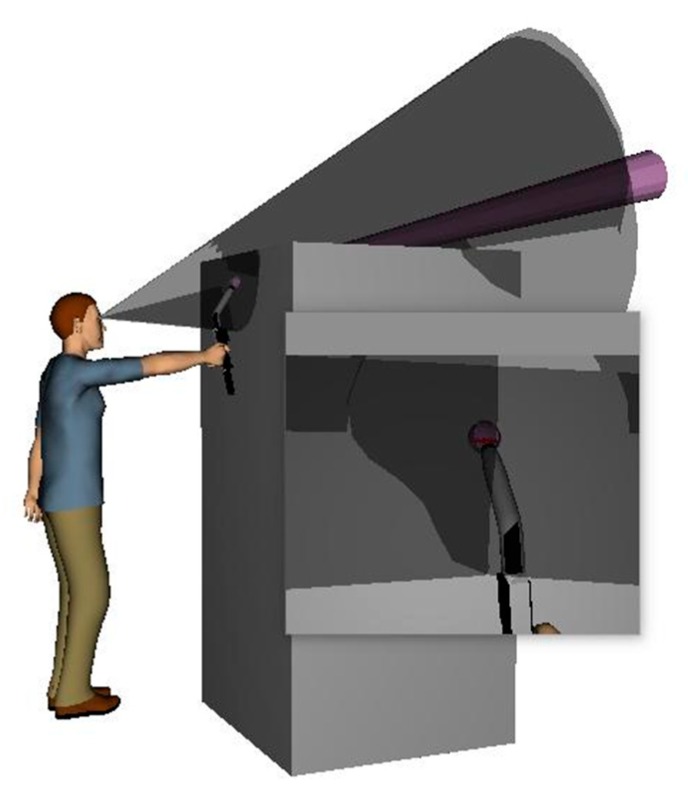
Emission particle and cone window.

The welder has to adjust the angle of his neck joints in order to be able to see the solder joints. In the Jack software, the Gilbert and Johnson collision method is used [22]. The collision detection technology emits a line of sight particle simulation to the solder joint according to the position of the human body viewpoint. As shown in Figure 2, it determines whether the target is occluded on the basis of particle and environmental collision [23].

• Field of view

Welding is a precise manual operation, wherein the welder’s eyes need to properly capture the exact position of the weld for the operation to be performed [24]. The best horizontal direct field of view (−15°–15°) and vertical direct field of view (−45°–15°) from the standard of vision and viewport division of the Chinese Control Center ergonomics design guidelines were used as the vision parameters to construct the field of view of this welding operation.

• Visual angle calculation

The quality of the line of sight was evaluated and the bending angle of the neck joint of the welder was obtained to obtain the adjustment strategy for the human neck posture. In this study, the best viewing angle (middle field of view) criterion was used, with the optimal top viewing angle *θ*_0_ as the scope of sight, *θ*_0_ is 45°. The angle *θ*_1_, *θ*_2_ were calculated as the horizontal plane viewing angle and the vertical plane viewing angle, respectively. On the basis of the collision detection technology, the particle collision range in the viewing angle calculation program written in the Jack Script programming language was converted into the values of the viewing angles *θ*_1_ and *θ*_2_, and the visibility was evaluated by comparing *θ*_1_, *θ*_2_, and *θ*_0_.

## 3. Results

### 3.1. Welding Torch Weight

The results of the welding torch weight were obtained by using the Jack software to analyze the lower back, comfort value, and Ovako Working Posture Analysis of different percentiles of the figure. The results are, as follows:

#### 3.1.1. Lower Back Assessment (LBA)

**Figure 3:**
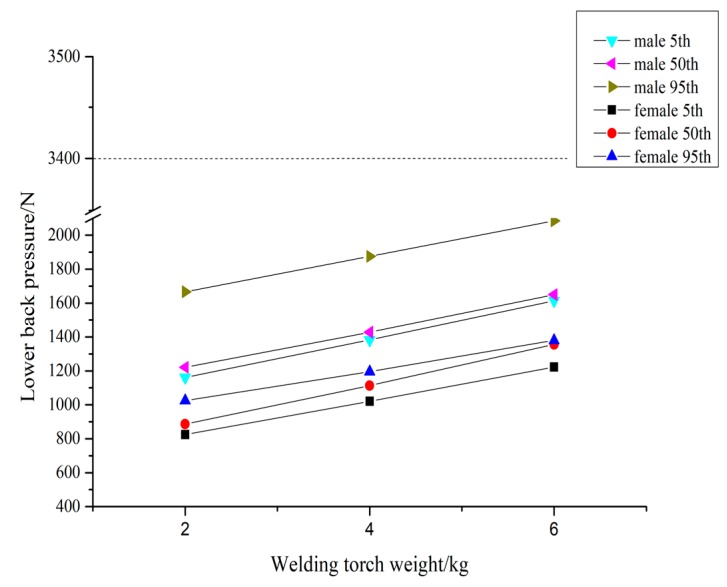
Lower back assessment of different welding torch weights.

The analysis of the welder’s animated process of lifting welding torches of 2 kg, 4 kg, and 6 kg was carried out to obtain the value of the lower back pressure, as shown in Figure 3. The 95th percentile of body size of female welders has the minimum value of LBA and the 95th percentile of male welders have the maximum value of LBA. The minimum value is 825 N and the maximum is 2084 N. Even if the maximum value is lower than the limit of 3400 N, as specified by NIOSH, it is reasonable for the welder exert that pressure on the lower back when lifting the conventional welding torch.

It can be seen from the results that the weight of the welding torch does not have a significant effect on the lower back pressure of different percentile humans, with gender being the main factor of stress. Therefore, for lightweight hand-held torches, their weight has little effect on the welder’s lower back pressure and it remains in a healthy range during operation.

#### 3.1.2. Comfort Assessment (CA)

The *MD*, *OV*, *HV*, and *LV* of each joint of each digital percentile of male and female welders were collected when measuring the maximum rotation angle of the human body. By substituting these values into Formula (1), the comfort of the corresponding joint can be calculated (while using the right hand-held welding torch as the prototype to measure the right half of the body joint). Table 4 shows the measurement results of the joint comfort of male and female welders, in this case, using a 6 kg hand-held welding torch.

The lower the calculated comfort value, the easier it is to represent the joint. According to the calculation results in the above table, in the right hand-held welding torch posture, some joints have an uncomfortable reaction, in which the right knee and the right torso and thigh generally feel discomfort, and for the right foot and the lower leg, females experience slight discomfort, while males do not.

#### 3.1.3. Ovako Working Postures Analysis (OWAS)

The conclusion of the analysis is that, regardless of the posture of both male and female welders, when using a welding torch of 6 kg or less, the evaluation grade that is given by the OWAS analysis is grade 2, that is, the working posture has a low risk of damage to the musculoskeletal system. Therefore, the effect of the weight of the welding torch on the working posture is acceptable.

### 3.2. Welder Upper Limb Posture

In the RULA, the upper limb load is less than 2 kg in order to avoid interference of the upper limb load on the upper limb posture itself. Before the simulation assessment of the upper limbs, a verification experiment was carried out. The verification experiment was intended to ensure that the computer model in the virtual environment accurately simulates the actual operation. The simulation processing time and actual operation time were compared in the study. The results show that, in standing welding operations, the difference between the simulated and actual exercise time of the standing walking action module was 0.98%; the difference between the simulated and actual exercise time of the standing raising arm module was 0.91%; the difference between the simulation actual exercise time of the standing contracting arm action module was 0.93%; with the maximum difference being less than one second. According to the comparison results, the simulation animation can simulate the actual operation well.

#### 3.2.1. Welder Operating Distance

**Figure 4:**
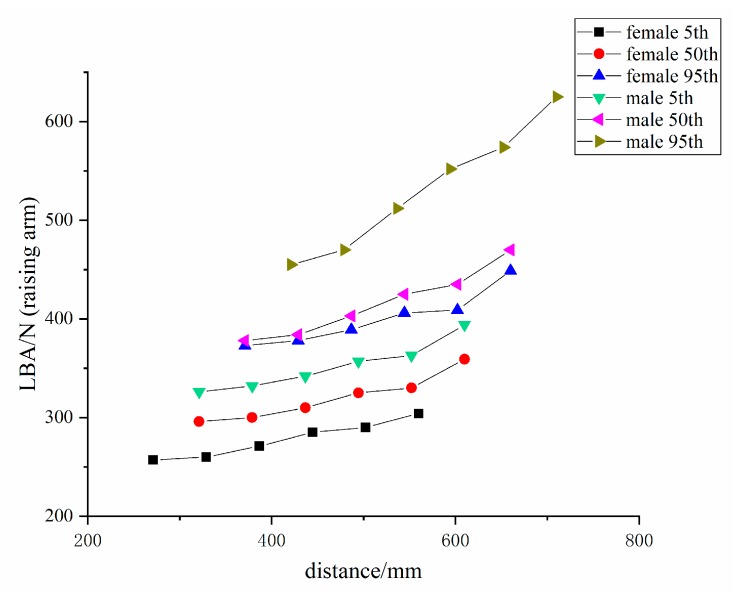
Lower back pressure at different operating distances for the standing raising arm action.

**Figure 5:**
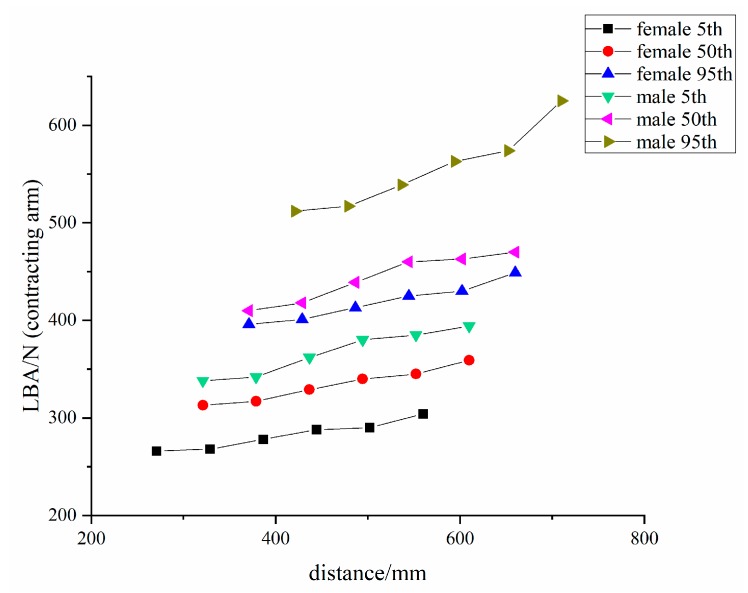
Lower back pressure at different operating distances for the standing contracting arm action.

This part of the study establishes three sets of work animations: standing walking, standing raising arm, and standing contracting arm. The welder operating distance is measured under the limit of the minimum and maximum functional rotation radius of the arm (hand) under the two sets of action modules: the standing raising arm and standing contracting arm. The 5th, 50th, and 95th percentile of body size for both males and females, a total of six digital human models, were used for LBA and RULA testing at different operating distances. Figure 4 and Figure 5 how the results.

It can be seen from the simulation results that, whether it is the standing raising arm action or the standing contracting arm action, the greater the operating distance, the greater the pressure on the lower back. In short, there is the positive correlation trend between the lower back pressure and operating distance. Therefore, according to the original design, for 5th, 50th, and 95th percentile males, the best operating distance is 321 mm, 371 mm, and 421 mm, respectively; and, for 5th, 50th, and 95th percentile females, the optimal operating distance is 271 mm, 321 mm, and 371 mm, respectively.

In the RULA scoring system of the Jack software, the higher the score, the greater the effect of the posture on the musculoskeletal system of the upper limb joints. Table 5 shows the RULA scores at different operating distances.

As can be seen from the above table, the grand score is 3 or 4. A grand score of 3–4 belongs to the second level, which indicates that the posture might require improvement. The digital human RULA scores for the welder’s operating distance of 502.2 mm are all three points, and the digital human RULA scores for distances of more than 444.4 mm are four points, which indicates that the farther away the welder’s upper limbs are, the greater the force on the musculoskeletal system of the upper limbs. This is also a positive correlation trend, which is consistent with the conclusions of the LBA study.

#### 3.2.2. Welding Torch Height from the Ground

**Figure 6:**
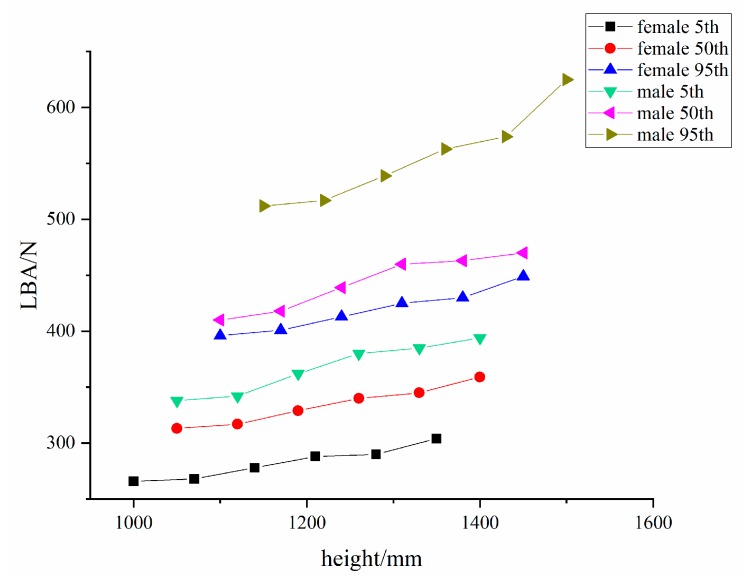
Lower back pressure of the welding torch at different heights from the ground.

Figure 6 shows the lower back pressure of the torch at different heights from the ground. According to the original design, the test was carried out for the most comfortable operating area for each percentile of digital person to obtain the optimum height from the ground.

While observing the graph, it can be seen that, in the comfort zone, when the human torso is standing upright and doing the lifting work, the higher the welder torch is from the ground, the greater the pressure on the lower back. That is to say that the height of the welding torch from the ground is positively related to the pressure on the lower back. Therefore, according to the original design, the best operating heights for the 5th, 50th, and 95th percentile males are 1050 mm, 1100 mm, and 1150 mm, respectively. For females in the 5th, 50th, and 95th percentile, the optimal operating heights are 1000 mm, 1050 mm, and 1100 mm, respectively.

Table 6 shows the RULA scores for different heights of the welding torch from the ground. The digital human RULA scores are all three points for heights under 1210 mm, while when the height is over 1280 mm, the digital human RULA scores are four points. This indicates that, in the upper limb comfort zone, the farther away the welding torch is from the ground, the greater the pressure on musculoskeletal system of the upper limbs, which is also a positive correlation trend, and it is again consistent with the conclusion of the LBA study.

### 3.3. Welder Neck Posture

The digital person, which is based on the best field of view, as mentioned previously, was selected, and the collision detection technology during the welding operation was used to test the range of the angle of view of the digital human for both the horizontal and vertical angle of view *θ*_1_, *θ*_2_.

**Figure 7:**
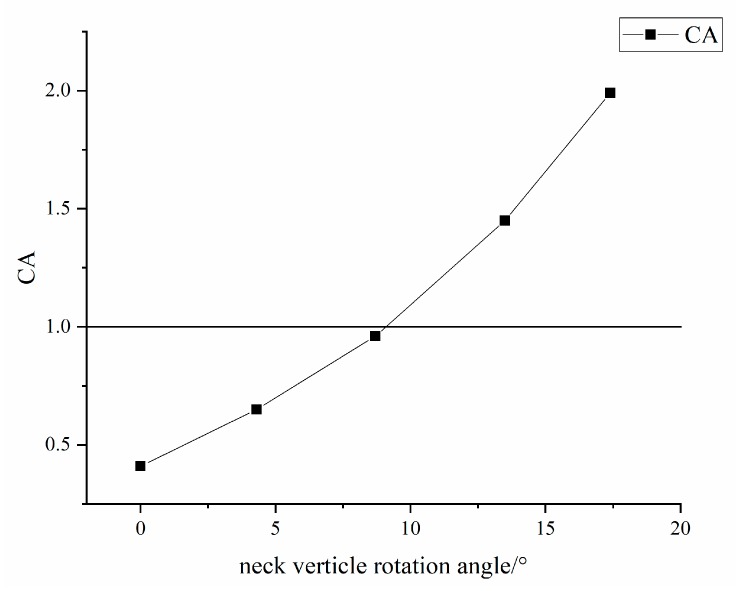
Comfort value changes with the angle of vertical deflection of the neck joint.

In manual work that requires precise operation for prolonged periods, in order to ensure the comfort of the human eye, the eyeball is usually maintained in a constant state of direct viewing [25]. Therefore, it is assumed that the welder’s eyeball is also kept stationary during the welding operation, and the adjustment of the viewing angle can only be adjusted by the neck joint. Therefore, the welder’s viewing angle *θ*_1_∈ [−15°, 15°], *θ*_2_∈ [−8.7°, 0°] is the adjustment range of the human neck joint. According to the angle of view of the welder, it is judged that the reasonable horizontal angle of rotation of the welder’s neck should not exceed 15° and the vertical rotation angle should not exceed 8.7°. This can be obtained by calculating the CA value at different neck joints at the vertical twist angle. When *θ*_2_ > 8.7°, the CA value significantly increases, and when it is greater than 1, the neck feels uncomfortable, as shown in Figure 7. Therefore, when the welder is in a standing welding position, the reasonable horizontal rotation angle of the neck should not exceed 15°, and the vertical rotation angle should not exceed 8.7°.

## 4. Discussion

When compared with traditional ergonomics evaluation methods, the Jack software simulation analysis that was used in this study combined with actual human body verification during operation provides a more comprehensive ergonomic evaluation method, allowing for users to create various types of welding environments. The Jack software uses a modeled digital human body, meaning that the participation of real welders is not required, which greatly reduces the cost of testing. The experimental results that were generated by the software avoid the complex environmental variables associated with the experiment and also avoid the interference and influence of the behavior of the welder before and after the operation. Therefore, the research on safety ergonomics that is based on digital human body modeling using Jack software is economic and scientific.

Research on the weight of the welding torch shows that the weight of the welding torch should not exceed 6 kg. Cao W et al. showed that, in the evaluation of the ergonomics of the lower limbs in hospital nurses, when an empty stretcher (less than or equal to 6 kg) was lifted, the pressure of each part of the human body was within the prescribed allowable stress range; however, when a patient is lifted (greater than 6 kg), the pressure exceeds the limit [26]. Similarly, Vieira used a questionnaire to review injury records, assessing the work-related lower back injuries of 64 welders, and comparing the discomfort scores and visual analogues with one-way ANOVA and Fisher’s least significant difference post-test. The results show that the average weight of the welder’s manual operation is 6 kg [27]. The findings of this are similar to those of the safety ergonomics study in this paper.

The upper limb posture study shows that, according to the percentile of body size of Chinese welders, the best operating distances for males in the 5th, 50th, and 95th percentile are 321 mm, 371 mm, and 421 mm, respectively, and the optimal operating heights are 1050 mm, 1100 mm, and 1150 mm, respectively; for females in the 5th, 50th, and 95th percentile, the optimal operating distances are 271 mm, 321 mm, and 371 mm, respectively, and the optimal operating heights are 1000 mm, 1050 mm, and 1100 mm, respectively. It can be seen from Table 4 that, during operation, the higher the percentile, the higher the comfort value, with the comfort value being greater than 1 with the increase in the percentile. This means that, the larger the person, the greater the stress on the limbs and joints, and the greater the discomfort. Figure 3, Figure 4, Figure 5 and Figure 6 indicate that, the higher the percentile, the greater the lower back pressure. Table 5 and Table 6 from the RULA score also indicate that taller people seem to be more likely to suffer from WMSDs. This is because taller workers do the same welding work, but their posture adjustment is larger and their discomfort is enhanced. This is consistent with previous research results, which state that the higher the body mass index (BMI), the greater the risk of developing WMSDs [28].

According to Figure 4 and Figure 5, when comparing the standing raising arm posture with the standing contracting arm posture, the latter exerts more force on the lower back at the same operating distance. Moreover, by comparing the six digital humans in the standing contracting arm operation of Figure 5, it was found that, when the operating distance is the same, the upper limbs are longer and the lower back is more stressed. This also proves that the curved upper limb posture causes greater lower back force when the operating distance is same. This conclusion confirms the results of using CATIA software to improve the research of hand-held dental devices [29].

Our study of neck posture cleverly applied visualization techniques. The rotation of the eyeball translates into the rotation of the neck under direct vision. The neck will feel uncomfortable when the horizontal rotation angle exceeds 15° and the neck vertical rotation angle exceeds 8.7°, according to our force analysis of the neck.

## 5. Conclusions

(1) From the study of the lower back pressure, comfort value, and upper limb force, it is not recommended for welders to use a welding torch weighing more than 6 kg.
(2) When considering the lower back pressure and upper limb force, for males in the 5th, 50th, and 95th percentile of body size, the optimal operating distances are 321 mm, 371 mm, and 421 mm, respectively, and the optimal operating heights are 1050 mm, 1100 mm, and 1150 mm, respectively. For females in the 5th, 50th, and 95th percentile of body size, the optimal operating distances are 271 mm, 321 mm, and 371 mm, respectively, and the optimal operating heights are 1000 mm, 1050 mm, and 1100 mm, respectively.
(3) The horizontal angle of rotation of the welder’s neck should not exceed 15° and the vertical angle of rotation should not exceed 8.7°.

## Figures and Tables

**Table 1:** Chinese localized welder body size parameters.

Dimension Name	Gender	Average Value	Standard Deviation	5th	50th	95th
Height/cm	M	168.7	5.6	159.5	168.7	177.9
	F	156.3	4.9	148.2	156.3	164.4
Weight/kg	M	67.3	8.6	53.1	67.3	81.5
	F	53.8	7.1	42.0	53.8	65.5
Maximum shoulder width/cm	M	45.4	3.4	39.7	45.4	51.0
	F	41.2	2.3	37.4	41.2	45.1
Sitting shoulder height/cm	M	33.4	1.8	30.5	33.4	36.3
	F	30.7	1.7	28.0	30.7	33.4
Sitting elbow height/cm	M	26.3	2.4	22.3	26.3	30.2
	F	25.4	2.3	21.6	25.4	29.2
Sitting deep/cm	M	42.4	2.6	38.2	42.4	46.6
	F	38.4	2.3	34.5	38.4	42.2
Sitting eye height/cm	M	78.6	2.9	73.7	78.6	83.4
	F	73.2	2.8	68.6	73.2	77.8
Sitting knee height/cm	M	51.6	2.7	47.2	51.6	56.0
	F	46.7	2.1	43.3	46.7	50.1

**Table 2:** Arm (hand) function radius of rotation range.

Gender	5th	50th	95th
M	321–610 mm	371–660 mm	421–710 mm
F	271–560 mm	321–610 mm	371–660 mm

**Table 3:** Comfort operating area height interval.

Gender	5th	50th	95th
M	1050–1400 mm	1100–1450 mm	1150–1500 mm
F	1000–1350 mm	1050–1400 mm	1100–1450 mm

**Table 4:** Right body comfort value results (hand-held 6kg welding torch).

Body Parts	M5th	M50th	M95th	F5th	F50th	F95th
Upper arm right flexion	0.22	0.54	1.17	0.52	0.92	1.34
Right elbow	0.37	0.48	1.22	0.21	0.51	1.52
Right torso and thigh	3.14	3.14	3.46	3.07	3.24	3.18
Right knee	2.52	2.51	2.52	2.23	2.23	2.23
Right foot, calf	0.75	0.68	0.77	1.33	1.14	1.08

**Table 5:** Rapid Upper Limb Assessment (RULA) scores for different operating distances.

Operating Distance/mm	Male RULA Score			Female RULA Score		
	5th	50th	95th	5th	50th	95th
271.0	-	-	-	3	-	-
328.8	3	3	3	3	3	3
386.6	3	3	3	3	3	3
444.4	3	3	3	3	3	3
502.2	4	4	4	4	4	4
560.0	4	4	4	4	4	4
610.0	4	4	4	-	4	4
660.0	-	4	4	-	-	4
710.0	-	-	4	-	-	-

**Table 6:** RULA scores for different heights of welding torch from the ground.

Height/mm	Male RULA Score			Female RULA Score		
	5th	50th	95th	5th	50th	95th
1000	-	-	-	3	-	-
1070	3	3	3	3	3	3
1140	3	3	3	3	3	3
1210	3	3	3	3	3	3
1280	4	4	4	4	4	4
1350	4	4	4	4	4	4
1400	4	4	4	-	4	4
1450	-	4	4	-	-	4
1500	-	-	4	-	-	-
